# Association Between Left Ventricular Global Longitudinal Strain and Hepatic Inflammation and Fibrosis in Metabolic Dysfunction-Associated Steatotic Liver Disease

**DOI:** 10.3390/diagnostics15233007

**Published:** 2025-11-26

**Authors:** Alberto Rodolpho Hüning, Vitor Emer Egypto Rosa, Diogo Silva Piardi, Daniara Viegas Rebelo Assis, Tainá Vanes Ferreira, Leonardo Griseli, Fabio Cañellas Moreira, Luiz Alberto De Carli, Carolina Rigatti Hartmann, Gabriela Perdomo Coral, Roney Orismar Sampaio, Marcelo Luiz Campos Vieira, Flávio Tarasoutchi, Paulo Ernesto Leães, Angelo Alves De Mattos

**Affiliations:** 1 Instituto do Coracao (InCor), Hospital das Clínicas HCFMUSP, Faculdade de Medicina, Universidade de São Paulo, São Paulo 05403-000, SP, Brazil; vitor.egypto@gmail.com (V.E.E.R.); sampaioroney@yahoo.com.br (R.O.S.); mluiz766@terra.com.br (M.L.C.V.); flavio.tarasoutchi@hc.fm.usp.br (F.T.); 2 Hepatology Postgraduation Program, Department of Gastroenterology and Hepatology, Federal University of Health Sciences of Porto Alegre (UFCSPA), Porto Alegre 90050-170, RS, Brazil; carolinahartmann@gmail.com (C.R.H.); gabrielacoral@ufcspa.edu.br (G.P.C.); angeloamattos@gmail.com (A.A.D.M.); 3 Santa Casa de Misericórdia de Porto Alegre, Porto Alegre 90020-090, RS, Brazil; dpiardi@gmail.com (D.S.P.); leonardogriseli@gmail.com (L.G.); canellasecocardio@gmail.com (F.C.M.); decarli@luizdecarli.com.br (L.A.D.C.); peleaes@gmail.com (P.E.L.); 4 Medicine Course, Federal University of Health Sciences of Porto Alegre (UFCSPA), Porto Alegre 90050-170, RS, Brazil; daniarare@ufcspa.edu.br (D.V.R.A.); taina.ferreira@ufcspa.edu.br (T.V.F.)

**Keywords:** metabolic dysfunction-associated steatotic liver disease (MASLD), cardiovascular disease, transthoracic echocardiogram (TTE), global longitudinal strain (GLS), MASLD progression, cardiovascular risk, inflammation, fibrosis

## Abstract

**Background/Objectives**: Cardiovascular disease is the leading cause of mortality in patients with metabolic dysfunction-associated steatotic liver disease (MASLD), which includes simple steatosis (metabolic dysfunction-associated steatotic liver, MASL), metabolic dysfunction-associated steatohepatitis (MASH), and fibrosis. This study aimed to evaluate the association between left ventricular (LV) systolic function, measured by global longitudinal strain (GLS), and liver inflammation and fibrosis in obese patients with MASLD undergoing preoperative evaluation for bariatric surgery. **Methods**: Intraoperative liver biopsies classified patients into four groups: non-MASLD; MASL; MASH; and MASH with fibrosis. Preoperative transthoracic echocardiography (TTE) was performed, and LV GLS was assessed using automated strain analysis. **Results**: Ninety-two patients were included: 13 non-MASLD, 34 MASL, 21 MASH, and 24 MASH with fibrosis. Although most patients had normal LV GLS, values were significantly lower in the MASH with fibrosis group compared to the MASL and non-MASLD groups (*p* = 0.011). In multivariate analysis adjusted for HDL cholesterol and LV mass, LV GLS was associated with inflammation and fibrosis (OR 0.784; 95% CI 0.637–0.965; *p* = 0.022). **Conclusions**: LV GLS was significantly lower in patients with MASH and MASH with fibrosis and was associated with hepatic inflammation and fibrosis in obese individuals undergoing bariatric surgery.

## 1. Introduction

The term metabolic dysfunction-associated steatotic liver disease (MASLD) has recently replaced nonalcoholic fatty liver disease (NAFLD) to encompass distinct subgroups, including metabolic dysfunction-associated steatotic liver (MASL), metabolic dysfunction-associated steatohepatitis (MASH), as well as fibrosis and cirrhosis [1]. MASLD is characterized by excessive hepatic triglyceride accumulation in the presence of at least one cardiometabolic risk factor, such as obesity, prediabetes or type 2 diabetes mellitus (T2DM), hypertriglyceridemia, low high-density lipoprotein (HDL) levels, and systemic arterial hypertension [2]. MASLD is currently the most prevalent chronic liver disease worldwide, posing major public health challenges and ranking among the leading causes of liver-related morbidity and mortality. It affects nearly one-third of the global population, with prevalence rising in parallel with obesity and T2DM [3]. Systemic insulin resistance and related metabolic disturbances underlie the shared pathophysiology of MASLD and extrahepatic complications, particularly cardiovascular disease (CVD) [4]. MASLD is likely an independent risk factor for cardiovascular (CV) morbidity and mortality, and CVD is the leading cause of death among patients with MASLD [5].

Atherosclerosis is more prevalent in MASLD and approximately doubles the risk of CV events compared with the general population [6]. Additionally, when T2DM is present, the CV risk increases twofold [7]. This risk escalates with disease progression, particularly in advanced stages of fibrosis, even after adjustment for other cardiometabolic factors [8]. Although cirrhosis is a major driver of liver-related mortality, fibrosis is also associated with CVD risk [9]. In the context of CVD, a transthoracic echocardiogram (TTE) plays a fundamental role in evaluating left ventricular (LV) function, and several studies have demonstrated associations between liver disease and both systolic and diastolic dysfunction [10,11,12,13]. Global longitudinal strain (GLS) is a measure of overall LV systolic function [14], that can detect early myocardial impairment and has been shown to be reduced in patients with MASLD [15,16,17,18,19]. However, a clear correlation between GLS and early myocardial impairment across MASLD subgroups has not been established [2,20,21].

Therefore, this study aimed to examine the association between LV GLS and hepatic inflammation and fibrosis in obese patients with MASLD undergoing preoperative evaluation for bariatric surgery, with biopsy confirmation, given that most prior studies used noninvasive diagnostics.

## 2. Materials and Methods

### 2.1. Study Design and Population

The study was conducted at the Obesity Treatment Center of a tertiary center specializing in obesity and CV disease in Porto Alegre, Brazil. Adults (≥18 years) with obesity, defined as a body mass index (BMI) ≥ 30 kg/m^2^, and an indication for bariatric surgery [22] were prospectively enrolled between August 2022 and September 2024. Preoperative CV evaluation, including TTE with GLS analysis, was performed within 1 year prior to surgery. Liver biopsy was obtained intraoperatively during bariatric surgery.

**Figure 1:**
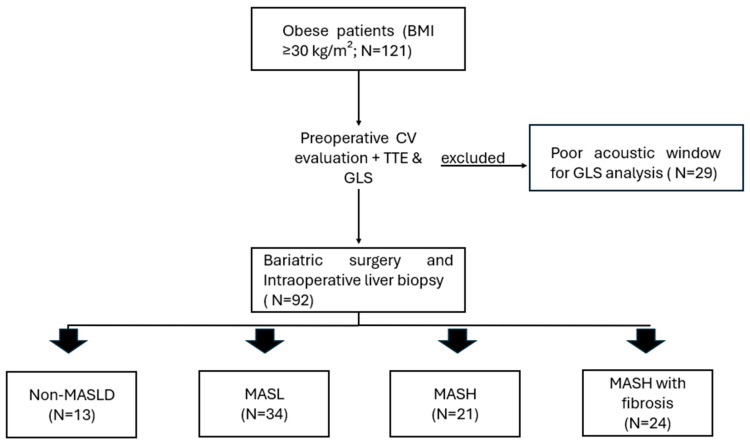
Study flowchart. Study flowchart and population selection. BMI, body mass index; CV evaluation, cardiovascular evaluation; TTE, transthoracic echocardiography; GLS, global longitudinal strain; non-MASLD, liver biopsy showing absence of steatosis; MASL, metabolic dysfunction–associated steatotic liver; MASH, metabolic dysfunction–associated steatohepatitis without fibrosis; MASH with fibrosis, metabolic dysfunction–associated steatohepatitis with fibrosis.

Exclusion criteria included inadequate acoustic windows for GLS analysis; prior use of hepatotoxic drugs; viral hepatitis (B or C); human immunodeficiency virus (HIV) infection; liver disease due to other chronic conditions (e.g., hemochromatosis, Wilson disease, autoimmune hepatitis, alpha-1 antitrypsin deficiency); alcohol consumption > 20 g/day for women or >30 g/day for men; hepatocellular carcinoma; history of angina and/or symptomatic coronary artery disease; pacemaker or defibrillator implantation; heart failure (HF) with reduced ejection fraction (<50%); and severe valvular heart disease or prior valve replacement surgery. The study adhered to the Declaration of Helsinki, was approved by the institutional ethics committee, and all participants were informed, clarified about the research, and provided written informed consent (Figure 1).

### 2.2. Data Collection

Clinical variables included age, sex, ethnicity, medical history (systemic arterial hypertension, T2DM, and smoking), medication use, blood pressure, and BMI. Preoperative laboratory testing included complete blood count (CBC), alanine aminotransferase (ALT), aspartate aminotransferase (AST), total cholesterol (TC), HDL, low-density lipoprotein (LDL), triglycerides (TG), glucose, glycated hemoglobin (HbA1c), and renal function estimated by the Chronic Kidney Disease Epidemiology Collaboration (CKD-EPI) equation. Obesity, systemic arterial hypertension, T2DM, and dyslipidemia were defined according to international guidelines [23,24,25]. Data were collected using the Research Electronic Data Capture (REDCap) electronic system [26].

### 2.3. Cardiovascular Evaluation

CV risk was assessed using three scoring systems: the Framingham Risk Score, the Atherosclerotic Cardiovascular Disease (ASCVD) score, and the American Heart Association’s Predicting Risk of Cardiovascular Disease Events (PREVENT) score, which accounts for the younger age of this population [27,28,29]. Electrocardiograms (EKG) data included the presence of arrhythmias (e.g., atrial fibrillation) and conduction abnormalities (right bundle branch block [RBBB]), left anterior fascicular block [LAFB], and first-degree atrioventricular block [AV block]. Corrected QT prolongation was evaluated, defined as >450 ms in men and >470 ms in women (calculated using the Bazzet, Hodges, and Framingham formulas). The assessment also included atrial enlargement, ventricular hypertrophy, and the presence of supraventricular and ventricular ectopic beats [30].

### 2.4. Transthoracic Echocardiogram and Global Longitudinal Strain Analysis

**Figure 2:**
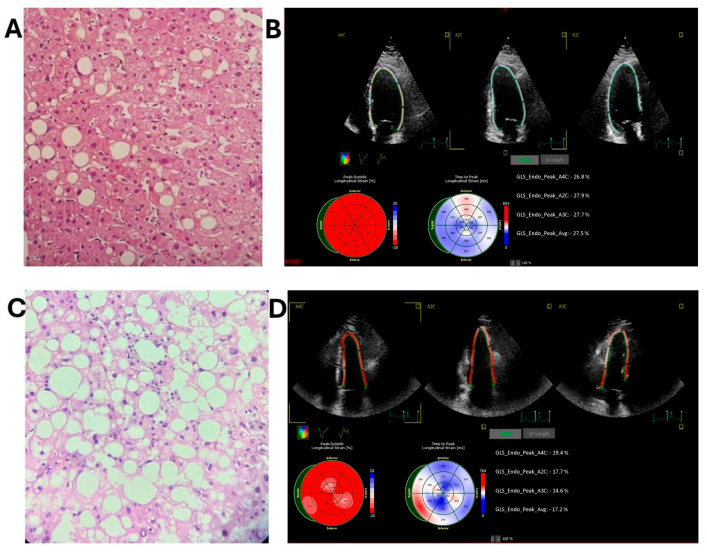
Histological images of MASLD diagnosed by liver biopsy and LV GLS results. (**A**) MASL case (HE stain showing hepatic parenchyma with macrovesicular steatosis). (**B**) MASL patient with LV GLS of 27.5% in apical 4-chamber (A4C), apical 2-chamber (A2C), and apical 3-chamber (A3C) views; longitudinal strain is measured at the endocardial border (green line). LV GLS is calculated as global endocardial shortening. (**C**) MASH case (HE stain showing hepatic parenchyma with >5% macrovesicular steatosis, hepatocellular ballooning, and inflammation). (**D**) MASH patient with LV GLS of 17.2% (A4C, A2C and A3C and longitudinal strain is measured at the endocardial border as indicated by the red line. GLS is calculated as global shortening of the endocardial border).

A commercially available system (Affiniti 70; Philips Medical Systems, Andover, MA, USA) was used to acquire standard apical four-chamber (A4C), two-chamber (A2C), and long-axis views, as well as a right-ventricle-focused four-chamber view. A minimum of four cardiac cycles was recorded per view, and images were archived in DICOM format. The LV end-diastolic volume (LVEDV), end-systolic volume (LVESV), and LV ejection fraction (LVEF) were calculated using the biplane Simpson method [14]. LV GLS was assessed by speckle-tracking echocardiography using AutoStrain, version 10.0 (TOMTEC, Unterschleissheim, Germany) for automated GLS analysis. After the evaluator selected the three apical views, the software automatically delineated the LV endocardial border using a knowledge-based artificial intelligence algorithm and performed speckle tracking over one cardiac cycle, generating regional strain curves and segmental strain values for each view (Figure 2B,D). The software then computed LV GLS [31]. All examinations were performed at the same institution within 12 months prior to surgery.

### 2.5. Metabolic Dysfunction-Associated Steatotic Liver Disease Diagnosis

The diagnosis of MASLD followed guidelines from the European Association for the Study of the Liver (EASL), European Association for the Study of Diabetes (EASD), and European Association for the Study of Obesity (EASO), requiring evidence of hepatic steatosis on imaging or histology and the exclusion of secondary causes of liver fat accumulation (e.g., significant alcohol intake, prolonged use of steatogenic medication, or monogenic disorders) [2]. Abdominal ultrasound was performed preoperatively as the initial diagnostic tool for MASLD.

Intraoperative liver biopsies were obtained from the left lateral segment using a cup biopsy grasper. Specimens were fixed in 10% formalin, embedded in paraffin, and stained with hematoxylin and eosin (HE), Prussian blue, and Masson’s trichrome, the latter two for the detection of iron and fibrosis, respectively. An experienced pathologist generated histopathological reports, classifying patients into four groups:

- Group 1—Non-MASLD: Liver biopsy showing absence of steatosis.
- Group 2—Metabolic dysfunction-associated steatotic liver (MASL) (Figure 2A): Liver biopsy demonstrating steatosis without signs of inflammation or fibrosis.
- Group 3—Metabolic dysfunction-associated steatohepatitis (MASH) without fibrosis (Figure 2C): Liver biopsy indicating steatohepatitis without fibrosis;
- Group 4—MASH with fibrosis: Liver biopsy demonstrating steatohepatitis with concurrent fibrosis.

Histological evaluation for MASL and MASH followed established criteria in the literature [32,33].

### 2.6. Statistical Analysis

Continuous variables were presented as median (interquartile range) and categorical variables as n (%). Normality was assessed with the Kolmogorov–Smirnov test. The Kruskal–Wallis test was used for continuous variables, and Fisher’s exact or chi-square tests for categorical variables, as appropriate. Post hoc comparisons used the Bonferroni test correction. Binary logistic regression was performed to predict inflammation/fibrosis on biopsy (Groups 3 and 4). Variables with *p* < 0.05 in univariable analysis were included in the multivariable model. Discriminative ability of LV GLS for advanced stages of MASLD was evaluated using receiver operating characteristic (ROC) curve analysis and the area under the curve (AUC). The LV GLS cutoff point was determined by the Youden index (J). A *p* < 0.05 level was considered statistically significant. Analyses were conducted using SPSS statistical software, version 21.

## 3. Results

### 3.1. Patient Characteristics

The main baseline clinical and laboratory data are presented in Table 1. A total of 121 patients were initially enrolled; 29 patients were excluded due to poor acoustic windows for LV GLS analysis, leaving 92 patients for analysis. Of these, 13 (14.1%) were classified as non-MASLD, 34 (36.9%) as MASL, 21 (22.8%) as MASH without fibrosis, and 24 (26%) as MASH with fibrosis. Most patients were female (84.8%), Caucasian (82.6%), with a median age of 38 (31–46) years, and predominantly obesity grade II–III (median BMI 39 (37–44) kg/m^2^).

Comorbidities were largely balanced across groups, with a higher prevalence of T2DM in the MASH with fibrosis group (29.2%) compared with MASH without fibrosis (4.8%), MASL (5.9%), and non-MASLD (7.7%) (*p* = 0.044), despite no differences in fasting glucose and HbA1c. Diastolic blood pressure differed among groups, with lower values in the non-MASLD group compared with MASL and MASH with fibrosis. Additionally, among patients with MASH, those without fibrosis exhibited lower blood pressure values (*p* = 0.012).

Preoperative abdominal ultrasound was normal in 22.8% of patients: 69.2% in non-MASLD, 29.4% in MASL, 9.5% in MASH without fibrosis, and 0% in MASH with fibrosis (*p* < 0.001). ALT was significantly higher in the MASH with fibrosis compared with the other groups (*p* = 0.011). No changes were observed in the patients’ clinical conditions or medication regimens during the interval between the echocardiogram with LV GLS analysis and the bariatric surgery procedure.

### 3.2. Cardiovascular Risk and Electrocardiogram Results

CV data are presented in Table 2. Based on Framingham, ASCVD, and PREVENT scores, 92.4%, 93%, and 96.7% of patients, respectively, were categorized as low risk; 6.5%, 3.3%, and 1% as intermediate risk; and approximately 1% as high CV risk by the Framingham score. All three scores indicated higher CV risk in the MASH groups (with and without fibrosis) compared to the non-MASLD. The PREVENT score also showed a higher risk estimate in MASL versus non-MASLD (*p* = 0.05). However, when stratified into 10-year CV risk categories (low, intermediate, and high), no difference was observed.

Regarding EKG evaluation (Table 3), all patients were in sinus rhythm, with heart rates ranging from 68 to 85 bpm. Intraventricular blocks were the most common findings, present in 47.8% of patients, primarily intraventricular conduction delay (IVCD) (44.6%). Left atrial enlargement was observed in 28.3% of cases. Ventricular escape beats occurred in 2.2%, and supraventricular ectopic beats in 1.1%. A prolonged QTc was noted in 5.4%. No between-group differences were observed in EKG parameters. (Table 3).

### 3.3. Echocardiographic Data and LV GLS Results

Echocardiographic parameters and LV GLS analysis are presented in Table 4 and Table 5. On TTE, 4.3% had diastolic dysfunction, 1.1% had systolic dysfunction, and 2.2% had LV GLS of ≤18%. Increased LV septal thickness and septal wall thickness were observed in both MASH groups compared with the non-MASLD group (*p* = 0.04 and *p* = 0.017, respectively). Relative LV wall thickness also differed among groups (*p* = 0.044).

Although most values were within normal limits, median LV GLS (%) was lower in the MASH with fibrosis group (22.4 (20.9–23.2)%) compared with MASL (24.2 (22.7–25.4)%) and non-MASLD (24.2 (22.8–25.2)%) (*p* = 0.011) (Table 5).

### 3.4. Discriminative Analysis and Mash Predictions:

**Figure 3:**
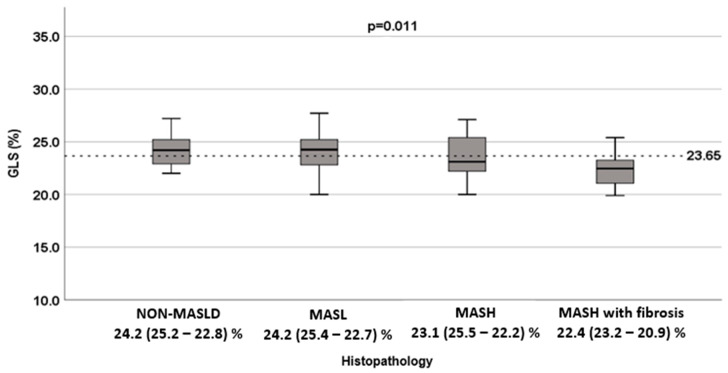
Comparison of LV GLS among non-MASLD, MASL, MASH, and MASH with fibrosis. Solid horizontal line indicates the mean; gray box, ±1 SD; vertical lines, minimum and maximum values. Non-MASLD: Liver biopsy showing absence of steatosis; MASL: Metabolic dysfunction-associated steatotic liver; MASH: Metabolic dysfunction-associated steatohepatitis without fibrosis; MASH with fibrosis: Metabolic dysfunction-associated steatohepatitis (MASH) with fibrosis.

The area under the ROC curve (AUC) for LV GLS to discriminate MASH with fibrosis was 0.66. An LV GLS threshold of 23.65% provided the highest accuracy, with a sensitivity of 63.8% and 71.1% specificity for discriminating the presence of inflammation and hepatic fibrosis (MASH with fibrosis), consistent with early myocardial impairment (Figure 3).

In multivariable analysis adjusted for HDL cholesterol and LV mass, LV GLS was associated with MASH with fibrosis (Odds Ratio: 0.784, 95% CI, 0.637–0.965; *p* = 0.022) (Table 6).

## 4. Discussion

The main findings of this study were: (1) most patients had normal LV GLS, but values were significantly lower in those with MASH with fibrosis (Group 4) compared with non-MASLD and MASL (Groups 1 and 2); and (2) LV GLS was associated with the presence of MASH and MASH with fibrosis (Groups 3 and 4).

MASLD prevalence is high worldwide, particularly in Latin America [3], affecting 69.9% of overweight individuals, with steatosis in 42.5% and MASH in 33.5%. Similar patterns are observed in obese populations, with 75.34% affected, 43.05% with steatosis, and 33.67% with MASH. Significant fibrosis was present in 20.27% of overweight and 21.60% of obese patients with MASLD [34]. In our cohort of patients with grade II–III obesity undergoing evaluation for bariatric surgery, MASLD prevalence was higher (85.8%), with lower MASL prevalence (36.9%) and higher prevalence of MASH (48.9%) and fibrosis (26%). The cohort was predominantly young, female, and Caucasian, reflecting a higher prevalence among women (84.8%) and a younger demographic compared with larger MASLD studies [3,35].

The principal novelty and key distinction of this study is the diagnosis of MASLD using the gold standard (histopathological confirmation), whereas most prior studies relied on non-invasive methods such as ultrasound [36,37]. Our findings align with the literature, underscoring the limitations of noninvasive diagnosis: only 70% of patients with MASL had concordant ultrasound findings, while 30% of those without MASLD showed steatosis.

The pathophysiology links between MASLD and CVD are multifactorial—encompassing genetic/epigenetic factors, endothelial dysfunction, dyslipidemia, insulin resistance, and gut microbiota alterations—which help explain the frequent coexistence of hypertension, T2DM, dyslipidemia, and metabolic syndrome [38]. All are prevalent in our cohort.

Hepatic fibrosis is associated with increased cardiovascular risk; however, specific predictive tools for cardiovascular outcomes in MASLD are lacking [39]. Existing risk scores perform suboptimally in individuals under 40 years [4,28,40]. Although the estimated 10-year cardiovascular risk was higher in the MASH groups (with and without fibrosis) versus non-MASLD, and higher in MASL versus non-MASLD by the PREVENT score, overall risk remained low (89–93%). This likely reflects the preoperative setting—requiring clinical optimization before bariatric surgery and the younger age of the cohort (median age of 38 (31–46) years), highlighting the limitations of current risk scores (ASCVD, Framingham, PREVENT) in young obese patients with MASLD.

The complex link between MASLD and CVD, specifically the action of cytokines originating from both systemic circulation and pericardial adipose tissue, may explain the development of atrial fibrillation and other cardiac arrhythmias, such as long QT syndrome [41,42,43]. The study population primarily exhibited minor EKG abnormalities. Although 5.4% of patients had prolonged corrected QT intervals, this finding did not correlate with the presence of MASLD, in contrast to previous studies [42,44].

MASLD is associated with the development of HF and ventricular remodeling, potentially resulting in diastolic and systolic dysfunction [10,11,12,13]. A retrospective study of approximately 174,000 patients reported a significantly higher 10-year incidence of HF in those with liver disease (13.2%) versus those without (10%) [45]. A meta-analysis of 31 studies including 40,000 patients with MASLD showed a higher prevalence of heart failure with preserved ejection fraction (HFpEF). MASLD was also associated with higher rates of diastolic dysfunction (up to 65%), increased indexed LA volume, and increased indexed LV mass [46]. In our study, echocardiographic abnormalities were relatively infrequent (4.3% for diastolic dysfunction and 1.1% for systolic dysfunction), likely reflecting the preoperative context and low CV risk profile of the cohort. Notably, despite remaining within normal limits, septal and posterior wall thicknesses were increased in the MASH groups. Indexed LV mass was associated with MASH in univariable but not multivariable analysis.

Global strain assesses myocardial deformation through three components of contraction: longitudinal, radial, and circumferential. Longitudinal deformation changes earliest, indicating subclinical dysfunction before any reduction in ejection fraction [47,48]. GLS is clinically relevant for prognostic stratification and for detecting early myocardial involvement in multiple settings—including LV hypertrophy, valvular heart disease, mechanical dyssynchrony, cardio-oncology, stable coronary artery disease, and acute coronary syndromes—though it has been less specifically studied in MASLD [49,50]. Emerging evidence shows that reduced GLS can reveal systolic dysfunction not detected by LVEF and offers superior prognostic value to LVEF for predicting major adverse cardiovascular events (MACE) across diverse cardiovascular conditions. GLS is associated with cardiovascular mortality and HF hospitalization in patients with preserved or mildly reduced LVEF. However, further research is needed to validate the prognostic role of GLS in HFpEF [50].

In MASLD, LV GLS may be significantly lower than in non-MASLD patients, as shown in a systematic review [15]. However, heterogeneity across studies must be considered. Karabay et al. [17] reported lower GLS in MASLD versus controls, but differentiation among MASLD subgroups was not possible. Notably, in normotensive, nondiabetic, nonobese patients with normal lipid profiles, no significant echocardiographic differences were observed compared with healthy individuals [17].

In obese patients with MASLD, GLS reduction may reflect coexisting conditions such as masked hypertension or subclinical obstructive coronary artery disease (CAD) [50]. In hypertension, reduced LV GLS indicates subclinical myocardial dysfunction and is often the earliest abnormality in stage A hypertension preceding HF; it can improve with long-term antihypertensive therapy [51,52,53,54]. Subclinical LV systolic dysfunction is also common in obstructive CAD despite normal LVEF, particularly in patients at higher atherosclerotic risk or with stable chest pain [50]. In diabetes, alterations in LV GLS may also detect subclinical myocardial dysfunction and may be associated with poorer glycemic control (HbA1c ≥ 8) [55,56]. Thus, LV GLS measurement is relevant in MASLD, a population commonly presenting with both hypertension and diabetes, affected by hypertension and diabetes, which also increases CAD risk. In our cohort, however, patients had well-controlled blood pressure and glycemia, no documented CAD, and only 1% were classified as having intermediate cardiovascular risk.

Although MASLD is associated with impaired systolic function independent of other risk factors, prior studies often relied on noninvasive diagnostic methods, as noted by Dong et al. in a diabetic population diagnosed via ultrasound [19]. Consistent with previous reports [36,37], our study highlights the limitations of ultrasound in obese patients: only 70% of individuals with MASL had findings concordant with the diagnosis, while 30% of non-MASLD patients showed steatosis on ultrasonography. This study distinguishes itself by confirming MASLD via biopsy in a highly specific cohort (obese individuals scheduled for bariatric surgery) and by evaluating GLS across distinct pathophysiological stages of the disease.

In this study, although a typical reduction in LV GLS among MASLD patients was not observed, the MASH with fibrosis group had significantly lower LV GLS than both the MASL group and non-MASLD patients. Despite the low proportion of men (15.2%), there is no strong evidence of sex-based differences in GLS values [31,57], and the number of male participants was similar across groups. The absence of patients with GLS < 18% in the fibrosis group may be explained by the highly specific cohort of obese individuals undergoing bariatric surgery, who underwent preoperative cardiovascular evaluation and thus likely represented a lower-risk cardiac profile than the general population. Moreover, many studies defining the normal GLS cutoff (≥18%) were validated in individuals with normal body weight. Body weight and the specific analysis software may influence GLS values; for example, TOMTEC AutoStrain tends to yield higher GLS measurements [31].

In an obese MASLD cohort undergoing bariatric surgery, a higher GLS discriminatory cutoff of 23.6% was identified. All patients with MASLD should undergo intensive evaluation and management of risk factors, in addition to cardiovascular risk assessment: lipid control with statins, smoking cessation, treatment of hypertension, glycemic control in type 2 diabetes, and weight loss with exercise [2]. Regarding the practical implications of reduced GLS, the interplay between CVD and MASLD suggests a potential role for SGLT2 inhibitors, including in advanced chronic liver disease. SGLT2 inhibitors have also shown promise in MASLD, significantly reducing hepatic steatosis and fibrosis in both diabetic and nondiabetic patients. Beyond glycemic control, these agents provide cardioprotective, pleiotropic benefits, including modulation of nutrient signaling, attenuation of oxidative stress and inflammatory pathways, improved mitochondrial function, strengthened antioxidant defenses, and enhanced endothelial integrity [58]. Therefore, performing sequential assessments of LV GLS within the same patient may be a promising and innovative approach for monitoring MASH and guiding appropriate treatment.

Limitations: The study included a relatively small number of patients, stratified into four groups, which may have influenced the results. However, the study’s novelty and key distinction from previous research is the diagnosis of MASLD using the gold standard (biopsy confirmation) in a highly specific population (obese individuals planning bariatric surgery), which makes enrolling a larger number of patients challenging. It is possible that if a larger number of patients were included, the results would achieve greater consistency. Although no changes in clinical status or medication were observed between the GLS echocardiogram and bariatric surgery, a further limitation is that echocardiography was performed up to 12 months before surgery. The relatively small number of male participants is also a limitation. However, there is no strong evidence of significant sex-based differences in GLS, and the number of male participants was similar across groups.

The study population consisted exclusively of obese patients with MASLD undergoing preoperative evaluation for bariatric surgery, potentially selecting for a subgroup with lower cardiovascular risk. Body fat distribution may be a determinant of the metabolic heterogeneity of obesity and its associated cardiovascular risk. This study did not assess the relationship between visceral and subcutaneous fat distribution and changes in GLS, which may represent a limitation. Furthermore, the cohort exhibited a high prevalence of comorbidities, which could have further affected the results, although most patients maintained controlled blood pressure and glycemic levels and had a low probability of CAD. Although a higher GLS discriminatory cutoff value of 23.6% was identified, the AUC reported indicates limited predictive power and represents an important limitation. The exploratory analysis included only variables significant in univariable testing in the multivariable model. Although factors such as age, metabolic risk, and cardiovascular comorbidities may still confound the associations, we emphasize the need for studies with more comprehensive multivariable adjustment. Despite this and the cross-sectional design, which precludes establishing causal relationships, we observed a progressive decrease in GLS in patients with more advanced MASLD subgroups. However, prognostic studies are needed to confirm these findings and their significance.

## 5. Conclusions

In obese patients undergoing bariatric surgery with biopsy-confirmed MASLD, LV GLS was significantly lower in individuals with MASH and MASH with fibrosis. This reduction was associated with the presence of inflammation and hepatic fibrosis. This study is hypothesis-generating, and if our findings are validated by further prospective studies with larger patient populations, LV GLS might become a useful non-invasive tool for stratifying individuals with MASLD. Such stratification would help identify those who might benefit from a more detailed evaluation of their cardiovascular risk profile and liver disease.

## Figures and Tables

**Table 1:** Clinical characteristics of the patients at baseline.

Variable	Total (n = 92)	Non-MASLD (n = 13)	MASL (n = 34)	MASH (n = 21)	MASH with Fibrosis (n = 24)	*p*
Clinical Data						
Age, years	38 (31–46)	36 (33–43)	38.5 (31–46)	44 (36–55)	35 (30–43)	0.194
Caucasian	76 (82.6)	9 (69.2)	28 (82.4)	16 (76.2)	23 (95.8)	0.190
Male sex	14 (15.2)	0	4 (11.8)	5 (23.8)	5 (20.8)	0.101
Active smoking	2 (2.2)	0	0	2 (9.5)	0	0.268
Previous smoking	13 (14.1)	3 (23.1)	5 (14.7)	3 (14.3)	2 (8.3)	0.268
Hypertension	36 (39.1)	6 (46.2)	15 (44.1)	5 (23.8)	10 (41.7)	0.430
Untreated hypertension	6 (6.5)	0	3 (8.8)	3 (14.3)	0	0.077
Dyslipidemia	75 (81.5)	8 (61.5)	26 (76.4)	21 (100)	20 (83.3)	0.576
Untreated dyslipidemia	66 (71.7)	8 (61.5)	23 (67.6)	17 (81)	18 (75)	0.576
Diabetes mellitus	11 (12)	1 (7.7)	2 (5.9)	1 (4.8)	7 (29.2)	0.044
Antihypertensive use	30 (32.6)	0 **^a^**	12 (35.3)	8 (38.1)	10 (41.7) **^a^**	0.009
ACEi/ARBs use	25 (27.2)	0	10 (29.4)	6 (28.6)	9 (37.5)	0.023
CCBs use	5 (5.4)	0	0 **^a^**	0	5 (20.8) **^a^**	0.003
BMI, kg/m^2^	39 (37–44)	42 (37.5–45)	38 (37–45.25)	41 (37.5–43.5)	39 (37–43.75)	0.710
Systolic BP, mmHg	120 (110–130)	110 (110–129)	120 (111.5–130)	125 (120–130)	120 (112.5–137.5)	0.262
Diastolic BP, mmHg	75 (70–80)	70 (60–77.5) **^a,b^**	80 (70–71.5) **^b,c,d^**	70 (60–70) **^c,d^**	80 (70–80) **^a,c^**	0.012
CKD-EPI, mL/min 1.73 m^2^	110.5 (99.2–118)	111 (101–114)	111.5 (98.7–118.2)	99 (88–113.5) **^a^**	114.5 (107.2–121.2) **^a^**	0.035
Glucose, mg/dL	93 (84–104.7)	85 (79–96)	91.5 (82.7–104.2)	94 (87.5–109)	94 (84.7–109.7)	0.225
HbA1c, %	5.5 (5.2–5.9)	5.3 (5.15–5.8)	5.5 (5.3–5.8)	5.3 (5.0–5.9)	5.6 (5.3–6.0)	0.651
Total cholesterol, mg/dL	190 (165.2–211.5)	164 (158.5–194.5)	191 (165.7–210.5)	196 (163–217.5)	190 (177–216.7)	0.274
HDL, mg/dL	45.5 (38–53)	49 (36.5–60.5)	48.5 (38.7–57)	42 (39.5–49)	44.5 (36.2–51.7)	0.375
LDL, mg/dL	114 (90.5–130)	102 (88.5–108.6)	116 (96.7–132)	118 (90.2–134.5)	113.5 (79.2–128)	0.340
Triglycerides, mg/dL	125 (97.2–177)	99 (78.5–152.5)	122.5 (87.5–166)	134 (107.5–200)	140 (106.5–210)	0.183
AST, mg/dL	23 (19–28)	21 (20–26)	23 (19–28)	24 (16.5–28)	23.5 (21–30.5)	0.314
ALT, mg/dL	27 (21–36)	22 (17–31) **^a^**	24 (20.7–31) **^b^**	27 (18.5–44.5) **^c^**	33 (27–46.7) **^a,b,c^**	0.011
Hematocrit, %	40.65 (38.8–42.4)	39.8 (38.4–41.8) **^b^**	39.7 (38.5–41.5) **^a^**	43.1 (40.1–46.1) **^a,b^**	40.95 (38.8–42.7)	0.022
Hemoglobin, mg/dL	13.75 (13.1–14.2)	13.9 (12.9–14.1)	13.5 (12.9–13.8) **^a^**	14 (13.6–15.6) **^a^**	13.75 (13.1–14.3)	0.017
Normal abdominal ultrasound	21 (22.8)	9 (69.2) **^a,b,c^**	10 (29.4)b	2 (9.5) **^c^**	0 **^a^**	<0.001

Continuous variables are presented as median (25th percentile–75th percentile), while categorical variables are reported as number (%). The definitions used are as follows: Non-MASLD: Liver biopsy showing absence of steatosis; MASL: Metabolic dysfunction-associated steatotic liver; MASH: Metabolic dysfunction-associated steatohepatitis; ACEi: Angiotensin-converting enzyme inhibitors; ARBs: Angiotensin receptor blockers; CCBs: Calcium channel blockers; BMI: Body mass index; BP: Blood pressure; CKD-EPI: Chronic Kidney Disease Epidemiology Collaboration equation; HbA1c: Glycated hemoglobin; HDL: High-density lipoprotein; LDL: Low-density lipoprotein; AST: Aspartate aminotransferase; ALT: Alanine aminotransferase. Identical letters (**a**, **b**, **c**, **d**) indicate groups with statistically significant differences.

**Table 2:** Cardiovascular risk in patients with and without MASLD.

Risk Score	Total (n = 92)	Non-MASLD (n = 13)	MASL (n = 34)	MASH (n = 21)	MASH with Fibrosis (n = 24)	*p*
**ASCVD, %**	1.1 (0.5–2.1)	0.4 (0.3–1.1) **^a,b,c^**	0.85 (0.5–1.5)	1.7 (0.9–2.8) **^b,c^**	1.4 (0.7–2.6) **^a^**	0.002
Low (<5%)	86 (93)	11 (84.6)	33 (97.1)	19 (90.5)	23 (95.8)	0.491
Intermediate (7.5–19.9%)	3 (3.3)	1 (7.7)	1 (2.9)	1 (4.8)	0	
High Risk (≥20%)	0	0	0	0	0	
**Framingham, %**	2.9 (1.7–5.0)	1.5 (1.1–2.3) **^a,b^**	2.9 (1.8–4.5)	4.5 (2–7.5) **^b^**	3.4 (1.9–5.6) **^a^**	0.011
Low (<10%)	85 (92.4)	12 (92.3)	32 (94.1)	20 (95.2)	21 (87.5)	0.333
Intermediate (10–20%)	6 (6.5)	0	2 (5.9)	1 (4.8)	3 (12.5)	
High Risk (>20%)	1 (1.1)	1 (7.7)	0	0	0	
**PREVENT, %**	0.8 (0.4–1.5)	0.3 (0.3–0.8) **^a,b^**	0.8 (0.3–1) **^b^**	1.3 (0.6–2.2) **^c^**	1.15 (0.4–1.8) **^a,c^**	0.021
Low (<5%)	89 (96.7)	12 (92.3)	32 (94.1)	21 (100)	24 (100)	0.516
Intermediate (7.5–19.9%)	1 (1)	0	1 (2.9)	0	0	
High Risk (≥20%)	0	0	0	0	0	

**Notes:** 10-year risk for CV disease estimation in the study population and subgroups, with continuous variables presented as median (25th percentile–75th percentile) and categorical variables as n (%). ASCVD: Atherosclerotic Cardiovascular Disease Risk Score; Framingham: Framingham Risk Score; PREVENT: The Predicting Risk of Cardiovascular Disease Events calculator; Non-MASLD: No metabolic dysfunction-associated steatotic liver disease; MASL: Metabolic dysfunction-associated steatotic liver; MASH: Metabolic dysfunction-associated steatohepatitis without fibrosis; MASH with fibrosis: Metabolic dysfunction-associated steatohepatitis (MASH) with fibrosis. The association of CV risk according to the scores was assessed using risk estimation as both continuous and categorical variables (low, intermediate, and high risk). Identical letters (**a**, **b**, **c**) indicate groups with statistically significant differences.

**Table 3:** Electrocardiogram results.

Variable	Total (n = 92)	Non-MASLD (n = 13)	MASLD (n = 34)	MASH (n = 21)	MASH with Fibrosis (n = 24)	*p*
Sinus Rhythm	92 (100)	-	-	-	-	-
Heart Rate (HR)	75 (68–85)	79 (68.5–87.5)	73.5 (60–82.5)	75 (68.5–83.5)	76.5 (70.25–85.75)	0.410
Intraventricular Blocks	44 (47.8)	3 (23.1)	21 (61.8)	8 (38.1)	12 (50.0)	0.083
Left Anterosuperior Fascicular Block (LAFB)	2 (2.2)	0	1 (2.9)	0	1 (4.2)	0.586
Right Bundle-Branch Block (RBBB)	1 (1.1)	0	0	0	1 (4.2)	0.437
First-Degree AV Block	1 (1.1)	0	1 (2.9)	0	0	0.570
Intraventricular Conduction Delay (IVCD)	41 (44.6)	3 (23.1)	20 (58.8)	8 (38.1)	10 (41.7)	0.129
Supraventricular Ectopic Beats	1 (1.1)	0	0	1 (4.8)	0	0.393
Ventricular Ectopic Beats	2 (2.2)	1 (7.7)	0	1 (4.8)	0	0.243
LAE	26 (28.3)	2 (15.4)	13 (38.2)	5 (23.8)	6 (25.0)	0.375
RAE	1 (1.1)	0	0	1 (4.8)	0	0.393
LVH	1 (1.1)	0	1 (2.9)	0	0	0.570
QT Interval (corrected)—Bazzett, ms	423 (403.5–446.75)	438 (414.5–464)	422.5 (400.75–443)	426 (407.5–446)	412 (402.75–452.25)	0.295
QT Interval (corrected)—Fredericia, ms	404 (389–427.75)	423 (396.5–449)	403 (388.25–429)	409 (391.5–423)	399.5 (382–428.5)	0.260
QT Interval (corrected)—Hodges, ms	405 (390.25–425)	419 (395.5–444.5)	406 (389.75–428)	407 (392–420)	397 (384–424.75)	0.271
Prolonged QT Interval	5 (5.4)	0	3 (8.8)	1 (4.8)	1 (4.2)	0.532

Notes: Electrocardiographic results in study population and subgroups, with continuous variables presented as median (25th percentile–75th percentile) and categorical variables as n (%). LAFB: left anterosuperior fascicular block; RBBB: right bundle-branch block; AV: atrioventricular block; IVCD: intraventricular conduction delay; LAE: left atrial enlargement; RAE: right atrial enlargement; LVH: left ventricular hypertrophy; QT interval: Represents the time from the start of the QRS complex to the end of the T wave. Non-MASLD: liver biopsy showing absence of steatosis; MASL: metabolic dysfunction-associated steatotic liver; MASH: metabolic dysfunction-associated steatohepatitis without fibrosis; MASH with fibrosis: metabolic dysfunction-associated steatohepatitis (MASH) with fibrosis.

**Table 4:** Echocardiographic assessment in patients with and without MASLD.

Variable	Total (n = 92)	Non-MASLD (n = 13)	MASL (n = 34)	MASH (n = 21)	MASH with Fibrosis (n = 24)	*p*
Indexed Aortic Diameter, mm/m^2^	14 (13–16)	14 (12.5–15.5)	14 (13–16.2)	14 (13–16)	14 (13–15)	0.314
Indexed Left Atrial Volume, mL/m^2^	28 (25–32)	31 (28–33)	28 (25–32)	27.5 (23.2–31.7)	28 (24–30.7)	0.271
Right Ventricle Diameter, mm	26 (24–27)	26 (23.5–27)	25 (23.7–27)	26 (24–28)	25 (23.2–28)	0.682
Left Ventricle Diastolic Diameter, mm	50 (47–52)	50 (47.5–51.5)	52 (47–52)	49 (46–51.5)	50.5 (48–52.7)	0.574
Left Ventricle Systolic Diameter, mm	31 (29–33)	31 (28–33)	32 (28.7–33.2)	30 (29–33)	31 (30–34.5)	0.614
Septal Thickness, mm	9 (8–9)	8 (7.5–9) **^a,b^**	8 (8–9)	9 (8–10) **^a^**	9 (8–10) **^b^**	0.040
PPLV Wall, mm	8 (8–9)	8 (7.5–8) **^a,b^**	8 (8–9)	8 (8–9.5) **^a^**	9 (8–9) **^b^**	0.017
Relative Thickness of LV Wall	0.33 (0.31–0.36)	0.31 (0.3–0.34)	0.33 (0.3–0.36)	0.34 (0.32–0.37)	0.35 (0.32–0.37)	0.044
Indexed LV Diastolic Volume, mL/m^2^	55 (47.2–61)	55 (48–59)	57 (47.5–64.2)	53 (47.5–57.5)	55 (47–60.7)	0.443
Indexed LV Systolic Volume, mL/m^2^	18 (14.2–20.7)	17 (14–20.5)	19 (16–21.5)	17 (14–18.5)	17.5 (15–20.5)	0.459
Indexed LV Mass, g/m^2^	67 (59.2–76)	62 (57–68)	70 (59.7–76.2)	66 (57–78)	70 (60.2–75.7)	0.241
Ejection Fraction (%)	66 (64–69.6)	66 (65–67.5)	66 (64–70.2)	69 (64–71)	66 (64.2–69)	0.662
Diastolic Dysfunction, n (%)	4 (4.3)	0	1 (2.9)	1 (4.8)	2 (8.3)	0.557
Systolic Dysfunction, n (%)	1 (1.1)	0	0	0	1 (4.3)	0.426

Notes: Echocardiographic variables are presented as median (25th percentile–75th percentile) and categorical variables as n (%); Indexed Aortic, Left Atrial, LV Diastolic, Systolic, Mass: Measurements indexed by body surface area (m^2^); PPLV: Posterior Wall of the LV in mm; LV: Left Ventricle. Categorical measures are reported as n (%); Non-MASLD: Liver biopsy showing absence of steatosis; MASL: Metabolic dysfunction-associated steatotic liver; MASH: Metabolic dysfunction-associated steatohepatitis without fibrosis; MASH with fibrosis: Metabolic dysfunction-associated steatohepatitis (MASH) with fibrosis. Identical letters (**a**, **b**) indicate groups with statistically significant differences.

**Table 5:** Left Ventricle GLS analysis.

Global Longitudinal Strain (GLS)	Total (n = 92)	Non-MASLD (n = 13)	MASL (n = 34)	MASH (n = 21)	MASH with Fibrosis (n = 24)	*p*
GLS, %	23.4 (22.2–25.1)	24.2 (22.8–25.2) **^a^**	24.2 (22.7–25.4) **^b^**	23.1 (22.2–25.5)	22.4 (20.9–23.2) **^a,b^**	0.011
GLS ≤ 18%	2 (2.2%)	0	1 (2.9%)	1 (4.8%)	0	0.531

Notes: Left ventricular (LV) global longitudinal strain (GLS) presented as median (25th–75th percentile). GLS ≤ 18%: number of patients with reduced LV GLS according to the reference value recommended by the software. Categorical variables are presented as n (%). Non-MASLD: liver biopsy showing absence of steatosis; MASL: Metabolic dysfunction-associated steatotic liver; MASH: Metabolic dysfunction-associated steatohepatitis without fibrosis; MASH with fibrosis: Metabolic dysfunction-associated steatohepatitis (MASH) with fibrosis. Identical letters (**a**, **b**) indicate groups with statistically significant differences.

**Table 6:** Univariate and multivariate analysis for MASH predictors.

	Univariable				Multivariable			
		95% (CI)				95% (CI)		
	OR	Lower	Upper	*p*	OR	Lower	Upper	*p*
LV GLS, %	0.757	0.614	0.932	0.009	0.784	0.637	0.965	0.022
HDL, mg/dL	0.962	0.930	0.996	0.028	0.969	0.934	1.006	0.099
LV mass, g/m^2^	1.015	1.000	1.030	0.044	1.011	0.995	1.028	0.164

Notes: LV GLS: left ventricular global longitudinal strain; HDL: high-density lipoprotein; LV mass: left ventricular mass; CI: confidence interval; OR: odds ratio.

## Data Availability

The partial or complete deidentified data set can be made available to researchers seeking collaboration upon appropriate ethical approval. Potential collaborators may reach out to Alberto Rodolpho Hüning at arhuning@gmail.com or Leonardi Griselli at leonardogriselli@gmail.com following publication.

## Abbreviations

The following abbreviations are used in this manuscript:

A2C	apical 2-chamber
A3C	apical 3-chamber
A4C	apical 4-chamber
ASCVD	Atherosclerotic Cardiovascular Disease Risk Score
AST	aspartate aminotransferase
ALT	alanine aminotransferase
AUC	the area under the ROC curve
AV block	atrioventricular block
BMI	body mass index
BP	blood pressure
CAD	coronary artery disease
CBC	complete blood count
CI	confidence interval
CKD-EPI	Chronic Kidney Disease Epidemiology Collaboration
CVD	cardiovascular disease
CV	cardiovascular
EASD	European Association for the Study of Diabetes
EASL	European Association for the Study of the Liver
EASO	European Association for the Study of Obesity
EKG	Electrocardiograms
GLS	global longitudinal strain
HBa1c	Glycated hemoglobin
HDL	high-density lipoprotein
HF	heart failure
HFPEF	heart failure with preserved ejection fraction
HIV	human immunodeficiency virus
IVCD	intraventricular conduction delay
LAE	left atrial enlargement
LAFB	left anterosuperior fascicular block
LDL	low-density lipoprotein
LV	left ventricular
LVEDV	LV end-diastolic volume
LVEF	LV ejection fraction
LVESV	LV end-systolic volume
LV GLS	LV global longitudinal strain
LVH	left ventricular hypertrophy
MACE	major adverse cardiovascular events
MASH	metabolic dysfunction-associated steatohepatitis without fibrosis
MASL	metabolic dysfunction-associated steatotic liver
MASLD	metabolic dysfunction-associated steatotic liver disease
NAFLD	nonalcoholic fatty liver disease
Non-Masld	liver biopsy showing absence of steatosis
OR	odds-ratio
PREVENT	The Predicting Risk of Cardiovascular Disease Events calculator
PPLV	Posterior Wall of the LV
RAE	right atrial enlargement
RBBB	right bundle branch block
REDCap	Research Electronic Data Capture electronic system
RV	right ventricle
T2DM	type 2 diabetes mellitus
TC	total cholesterol
TG	triglycerides
TTE	transthoracic echocardiography
